# The Quest to Find True ADHD: Navigating the Diagnostic Challenge of Malingering – A Systematic Review

**DOI:** 10.1192/j.eurpsy.2025.622

**Published:** 2025-08-26

**Authors:** G. Ratti, A. Callari, G. Nosari, A. R. Bianchi, G. Delvecchio

**Affiliations:** 1 Department of Pathophysiology and Transplantation, University of Milan; 2 Department of Neurosciences and Mental Health, Fondazione IRCCS Ca’ Granda Ospedale Maggiore Policlinico, Milan, Italy

## Abstract

**Introduction:**

Attention-Deficit/Hyperactivity Disorder (ADHD) is a prevalent neurodevelopmental disorder and accurate diagnosis is essential but remains challenging due to the potential for malingering. Additionally, the increasing demands of a fast-paced society place, prompting quick solutions, may lead to simulated presentations or feigning. This paper examines the complexities of distinguishing genuine ADHD cases from malingering, emphasizing the need for refined diagnostic protocols. Malingering, in fact, involves deliberately fabricating symptoms to achieve a desired result, such as:

- **Academic Advantages**
- **Access to Medications**
- **Avoiding Responsibilities**

**Objectives:**

Given all these challenges, we explored how current scientific literature addresses this issue. Inspired by the work of many colleagues, we aimed to summarize their efforts in this systematic review to propose a possible solution for collaborative improvement.

**Methods:**

We analyzed over 296 papers from major scientific databases focusing on “malingering and ADHD” to create a comprehensive overview of this increasingly widespread problem.

**Results:**

The main results, after a thorough analysis of all parameters, emphasize the need for:

1. Advanced Assessment and Objective Measures: dynamic assessment techniques, combined with psychophysiological measures and parental input.

2. Enhanced Diagnostic Protocols and Tools Integration: ADHD rating scales and Symptom Validity Tests (SVTs) like TOMM (Test of Memory Malingering) and WMT (Word Memory Test) to minimize diagnostic errors, make the criteria more robust.

The following table summarizes the criteria analyzed in the 37 articles selected after a thorough review.

**Table:** 

Study Reference	Participants	Age	ADHD Assessment Tools	Malingering Assessment Tools	Comorbidities	Main Findings
Selected studies (37)	Diverse populations such as college students and ADHD patients.	Mean ages ranging from early 20s to late 20s; gender distribution varies.	Various scales including CAARS, BAARS-IV, ImPACT.	Several scales, including NV-MSVT, PAI, b Test, WMCD.	Commonly reported: anxiety and depression.	Challenges in differentiation, high prevalence of comorbidities, issues with malingering detection.

**Conclusions:**

Traditional ADHD assessments, based heavily on self-reported symptoms, are becoming less reliable, especially with the growing trend of self-diagnosis and malingering. To address this, adding performance-based tests like TOMM and WMT is key for spotting malingering and improving diagnosis accuracy. Our study aims to elucidate this issue, which has subtle but profound implications for the quality of life of thousands of patients, who may receive a superficial diagnosis that could affect them for a lifetime, even pharmacologically.

**Disclosure of Interest:**

None Declared

